# Prevalence and distribution of adhesins and the expression of fibronectin-binding protein (FnbA and FnbB) among *Staphylococcus aureus* isolates from Shahrekord Hospitals

**DOI:** 10.1186/s13104-019-4055-0

**Published:** 2019-01-22

**Authors:** Emad Soltani, Effat Farrokhi, Behnam Zamanzad, Milad Shahini Shams Abadi, Fatemeh Deris, Amin Soltani, Abolfazl Gholipour

**Affiliations:** 1 Cellular and Molecular Research Center, Basic Health Sciences Institute, Shahrekord University of Medical Sciences, Shahrekord, Iran; 2 Department of Bacteriology & Virology, School of Medicine, Shiraz University of Medical Sciences, Shiraz, Iran; 3 Department of Epidemiology and Biostatistics, School of Health, Shahrekord University of Medical Sciences, Shahrekord, Iran; 4 Medical Plants Research Center, Basic Health Sciences Institute, Shahrekord University of Medical Sciences, Shahrekord, Iran; 5 Department of Microbiology and Immunology, Cellular and Molecular Research Center, Shahrekord University of Medical Sciences, Shahrekord, IR Iran

**Keywords:** Staphylococcus aureus, Real-time PCR, Colonization

## Abstract

**Objective:**

One of the most important causes of nosocomial infections is *Staphylococcus aureus*. The aim of this study was to determine the frequency of these genes and the rate of expression of these genes during nasal colonization among the personnel of Kashani and Hajar hospitals.

**Results:**

In this Analytical-descriptive study, 240 nasal swab specimens were collected from personnel of different departments of Kashani and Hajar hospitals in Shahr-e-kord. Nasal specimens were cultured and 110 *Staphylococcus* strains were isolated. Based on the results, 110 carriers of *Staphylococcus aureus* were identified. The frequency of clfA, clfB, fnbA and fnbB genes were 36.3%, 86.3%, 7.2% and 43.6% respectively. It was also observed that the fnbA gene showed no expression, but of 95 clfB-positive samples, 73 isolates (76.8%) were expressed clfB gene. This study showed that the abundance of these genes varies in nasal colonization. It was also observed that clfB gene with a high frequency and high expression rate has an important role in nose colonization. These results not only provide insight into the factors involved in *S. aureus* colonization but also provide potential therapeutic targets.

## Introduction

*Staphylococcus aureus* is an important human pathogen and is colonized in skin and mucous membrane. This bacterium is permanently colonized in 20% of healthy adults in nose and skin [1]. People who are predisposed to nasal colonization with *S*. *aureus* are at the risk of infections such as mild skin and soft tissue infections, and also more serious diseases such as endocarditis, bacteremia, and sepsis [2, 3]. For many years, this bacterium has been considered a main hospital pathogen. It causes a wide range of epidermal infections, septicemia, endocarditis, and pneumonia that are life-threatening [4]. Important bacterial virulence factors are surface factors that cause bacteria to adhere and colonize at the surfaces. These specific adhesive agents can bind to a variety of host proteins, particularly those in the extracellular matrix (ECM), and also to the host cells. This binding is mediated by a family of proteins called microbial surface components recognizing adhesive matrix molecules (MSCRAMMs) [5, 6]. *S*. *aureus* can express various MSCRAMMs including *clfA, clfB, fnbA, fnbB, fib, Eno, can, ebps*, and *bbp* [7, 8]. ClfA (Clumping Factor A) is a staphylococcal fibrinogen-binding protein that is responsible for the accumulation of this bacterium in the blood plasma and plays an important role in arthritis and endocarditis. This factor also binds to platelets [9]. The ability of *S*. *aureus* to bind to and colonize in the nasal epithelium is due to the expression of the clfB surface protein [10]. Fibronectin-binding proteins (FnbA and FnbB) are effective in tissue invasion in various pathological conditions such as eye keratitis, osteomyelitis, and replacement at the surfaces of medical devices. FnbA/B is also a mediator of signaling cell and actin cytoskeletal rearrangement [11]. The adhesion gene fnbA leads to cardiovascular disease and cardiovascular system infection through platelet activation and also causes adhesions to artificial implants in the body [5]. The identification of the genes involved in colonization has attracted the researchers’ attention [6]. The aim of this study was to compare the prevalence of clfA, clfB, fnbA, fnbB genes involved in colonization, as well as to compare the expression patterns of the clfB and fnbA genes.

## Main text

### Materials and methods

#### Specimen collection and isolation and identification of bacteria

In this analytical-descriptive study, 240 nasal swab specimens were collected from the staff of different wards of Kashani and Hajar Teaching hospitals in Shahrekord. The swabs were immediately transferred to the TSB broth environment and incubated for 24 h. Then, *S. aureus* isolates were identified by performing tests such as hot dyeing, colony morphology, and various biochemical tests such as colony morphology, catalase activity, mannitol salt agar, coagulase test, and DNase test.

#### DNA extraction and RNA extraction and cDNA synthesis

DNA extraction was performed by boiling method. Sterile swabs were wetted with 250 μl of nucleoside and specimens were taken from the anterior nasal cavity of the participants. Subsequently, the swab was placed in 1000 μl of Trizole and RNA extraction was performed according to the Trillocentrum protocol. Then, 1000 ng of RNA was used for the synthesis of cDNA by using RevertAid First Strand cDNA Synthesis Kit.

#### PCR

In this study, specific primers were designed to confirm the presence of the clfA, clfB, fnbA, and fnbB genes, as well as measuring the expression of the clfB and fnbA genes. Also a primer pair was designed for the reference gene. The characteristics of the primers are shown in Table 1.

**Table 1:** Specifications of designed primers

Gene	Primer sequence(5′ to 3′)	Product length (bp)
fnbAF	TGGTACTGATGAAGTTGATTTTAGAAC	101
fnbAR	CATTATCCCAAGTTAAGGTATATCCTC	
clfBF	CCGGTAGTAAATGCTGCTGTA	103
clfBR	CACTTTGATTAGGGTCAAATGTAGTC	
16sF	GGTCTGTAACTGACGCTGATGTG	68
16sR	GTGGACTACCAGGGTATCTAATCCT	
fnbBF	GGAGCGGCCTCAGTATTCTT	201
fnbBR	AGTTGATGTCGCGCTGTATG	
clfAF	CGCCGGTAACTGGTGAAGCT	314
clfAR	TGCTCTCATTCTAGGCGCACTT	

The PCR conditions included an initial denaturation at 95 °C for 7 min, followed by a 35-cycle amplification consisting of denaturation at 95 °C for 45 s, annealing for 20 s and extension at 72 °C for 30 s followed by final extension at 72 °C for 1 min. After completing the PCR, the PCR product was tested for the bands and the presence of the genes of interest on a 8% polyacrylamide gel. The annealing temperature for clfA, clfB, fnbA, fnbB, and 16S rRNA genes was 57 °C, 57 °C, 56 °C, 59 °C, and 58 °C, respectively.

#### Real-time

In order to perform Real-time PCR, 6 µl SYBR™ Green Master (TAKARA), 4 µl of nuclease–free water, 1 µl of cDNA, and 0.5 µl of primer of the desired gene at a concentration of 5 pM were added into Real-time tube caps. According to the temperature cycling program, the tube caps were placed in the Real-time PCR instrument to measure the mRNA expression of clfB and fnbA genes with 16S rRNA as internal control [12]. The Real- time PCR conditions included an initial denaturation at 95^º^ C for 10 min, followed by a 40-cycle amplification consisting of denaturation at 95 °C for 10 s, annealing at 60 °C for 20 s and extension at 72 °C for 30 s followed by Melting curves to verify qPCR product identity.

#### Statistical analysis

Statistical analysis were determined by the exact fisher and K_2_ test, using IBM SPSS Statistics 20software differences were considered significant if P values were less than 0.05.

### Results

#### Investigating the relationship between the gene and the parts of the hospital by the method PCR

In this study, 240 swabs of nose of personnel of Hajar and Kashani hospitals were obtained and 110 samples of *Staphylococcus aureus* were positive (45%) including 53 samples from Hajar Hospital and 57 from Kashani Hospital. The frequency of clfA, clfB, fnbA and fnbB genes were observed (36.3%), (86.3%), (7.2%), and (43.6%) respectively. The highest frequency of clfA gene was observed in ICU (69.2%) and operating room (54.5%). ClfB gene was highly prevalent in most parts. The highest frequency of fnbB gene was found in the operating room (90.9%) and pediatrics (87.5%). FnbA gene had a low frequency in most parts. The relationship between the gene and the part of hospital is shown in Table 2.

**Table 2:** Investigating the relationship between the gene and the parts of the hospital

Parts	fnbB Number (%)	fnbA Number (%)	clfB Number (%)	clfA Number (%)	Total Number (%)
Infectious	45.4 (5)	0 (0)	10 (83.3)	4 (33.3)	12
Dialysis	45.4 (5)	2 (18.1)	10 (90.9)	4 (36.3)	11
Pediatrics	87.5 (7)	0 (0)	7 (87.5)	4 (50)	8
Surgery room	90.9 (10)	1 (9.09)	9 (81.8)	6 (54.5)	11
CCU	27.2 (3)	0 (0)	10 (90.9)	2 (18.1)	11
Men's surgery	25 (3)	0 (0)	8 (66.7)	3 (25)	12
Women's orthopedic	18.1 (2)	1 (9.09)	10 (90.9)	4 (36.3)	11
Men's orthopedic	27.2 (3)	0 (0)	10 (90.9)	3 (27.2)	11
Burns	0 (0)	0 (0)	9 (90)	1 (10)	10
ICU	76.9 (10)	4 (30.7)	12 (3.92)	9 (69.2)	13
P-value	0.026*	0.042*	0.006*	0.026*	110

* In the table, the values for the presence of the gene are in the relevant section. P-value <0.05

#### Results of real time and investigating the expression of clfB and fnbA genes

**Fig. 1:**
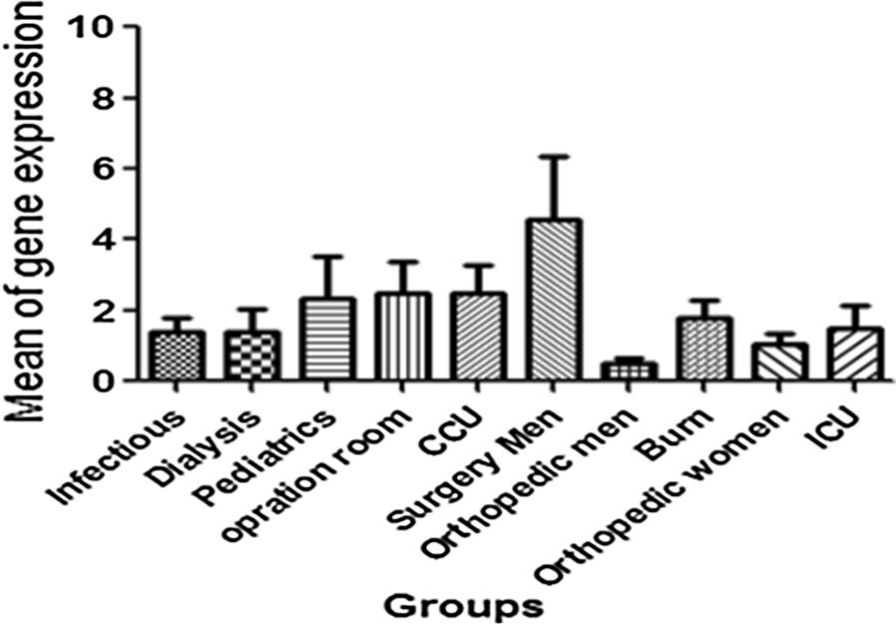
The average of clfB gene expression in each hospital segment. The average of clfB gene expression was compared in different parts of the population and there was no significant difference between the sections. P-value = 0.288

Out of 110 positive samples of *Staphylococcus aureus*, 8 samples contained the fnbA gene with no expression patterns. Of 95 samples contained clfB gene, 71 samples showed gene expression. The highest expression of the gene was related to bacteria isolated from the pediatric ward (100%) and the lowest expression of the gene belonged to the bacteria isolated from the dialysis section (50%). The average of clfB gene expression in each hospital segment is shown in Fig. 1.

### Discussion

*Staphylococcusaureus* is an important hospital pathogen, the most common cause of surgical wound infections, the second leading cause of hospital pneumonia, and also responsible for 35% of hospital-acquired bacteremia [3]. Among the many virulence factors of this bacterium, MASCRAMMs; responsible for the bacterial adhesion to the host, are more important [13]. In the current study, the coding genes for clfA and clfB, as well as the coding genes for the fibronectin-binding proteins A and B were investigated. In a total of 110 positive *S*. *aureus* specimens taken from the 10 wards of Hajar and Kashani Hospitals, the frequency of the clfA, clfB, fnbA, and fnbB genes was 36.3%, 86.3%, 7.2%, and 43.6%, respectively. The highest frequency in all wards was observed for the clfB gene with only 5 isolates lacking this gene. The lowest frequency was obtained for the fnbA gene with only 8 isolates containing this gene. *S*. *aureus* binds to cytokeratin 10 on epithelial cells with the help of the clfB, which increases the ability of the bacterium to bind and colonize, especially in the nose. For example, a study by Palmqvist et al. on Staphylococcal arthritis and systemic inflammation which 92% of the samples contained clfB gene, showed that clfB is involved in inducing local arthritis, as well as cartilage and bone destruction [14]. In another study by Salman sahab, Atshan et al. on 60 clinical specimens, all specimens were found to contain the clfB gene, and in a similar study on 36 clinical specimens, by Ghasemian et al. 100% of the specimens contained the clfB gene. In addition, in a study of 70 isolates from the people with bone infections by Rohde et al., the frequency of the clfB gene was reported to be 100% [6, 15, 16]. In a study by Ewa szczuka et al. which different clinical samples were collected over a period of 4 years, the results showed that the frequency of clfB gene in the isolates from patients with pneumonia was 97% and in those from patients with bacteremia, bone infection, skin and surface tissue infections, otitis media, and arthritis was 100% [17]. The results of all studies and the high prevalence of clfB gene indicated the remarkable role of this gene in the pathogenicity of *S*. *aureus*, as well as bacterial colonization at various surfaces. The results of our study are consistent with the results of the cited studies and confirms the role of this gene in the colonization in healthy individuals and patients. ClfA is also one of the bacterial virulence factors that bind to fibrinogen and contribute to arthritis and endocarditis, and can contribute to the accumulation of bacteria in the blood plasma [18]. In a study by Arciola on samples isolated from orthopedic infections, the frequency of the fnbA gene was 98% and the 99% fnbB gene was observed [19]. The influence of the clfA and fnbA genes on the pathogenicity of *S*. *aureus* in ear infections has been reported, with the frequency of 32% and 76%, respectively [20]. To determine the role of each of the clfB and fnbA genes in the colonization of *S*. *aureus* in healthy individuals, the expression of each of these genes was measured by Real-time PCR. Due to the high frequency of cytokeratin 10 receptors in the nose [21, 22] to which *S*. *aureus* binds to by clfB, the clfB gene expression is expected, as with its frequency, to be high. In our study, of the 95 clfB gene-containing specimens, 76.8% were observed to be expressed and none of the 8 samples containing the fnbA gene showed expression. The results obtained in this study suggest that *S*. *aureus* activates the clfB gene via its transcription and thus causes the bacterium to adhere to the nasal epithelial cells and colonize in there. Studies have shown that in addition to clfB, teichoic acid and isdA also play an important role in the binding and colonization of the nasal bacteria [23]. In a study by Jenkinz et al. on *S*. *aureus*-infected mice, fnbA gene expression was shown to increase during nasal colonization and in bacteremia and thromboembolic lesions [24]. On the other hand, other studies have shown that fnbA and TagO play a key role in binding to endothelial cells, which can indicate the comparatively less pronounced role of these genes in binding to epithelial cells [25–27]. Therefore, the results of this study are consistent with the results of the cited studies, indicating the role of the fnbA gene in the nasal colonization in vivo. In the study of Burian et al. on *S*. *aureus* samples during nasal colonization, the expression of the clfB gene was observed to increase [21]. This information on these issues can help to better understand the pathogenicity and also facilitation of the control and prevention of Staphylococcal infections. The inconsistency in the results of the present study and other studies regarding the expression of clfB and fnbA genes can be explained by the fact that clfB gene plays a *comparatively* more important and reportedly definite role in nasal colonization.

## Limitations

The lack of investigation and characterization of other virulence factors in *Staphylococcus aureus* isolates can be mentioned as one of the main limitations of the present study.
